# 类器官芯片在肺癌诊疗研究中的应用及进展

**DOI:** 10.3779/j.issn.1009-3419.2025.101.16

**Published:** 2025-09-20

**Authors:** Wuyang YUN, Xiaoyun ZHANG, Li XIAO

**Affiliations:** ^1^100091 北京，解放军总医院第八医学中心呼吸与危重症医学部，北京市器官移植与免疫调节重点实验室; ^1^College of Pulmonary and Critical Care Medicine, Beijing Key Laboratory of Organ Transplantation and Immunology Regulatory, The 8^th^ Medical Centre of Chinese PLA General Hospital, Beijing 100091, China; ^2^075000 张家口，河北北方学院; ^2^Hebei North University, Zhangjiakou 075000, China

**Keywords:** 肺肿瘤, 类器官, 类器官芯片, 免疫治疗, 抗肿瘤药物筛选, Lung neoplasms, Organoid, Organoid-on-a-chip, Immunotherapy, Antitumor drug screening assays

## Abstract

肺癌是全球范围内发病率和死亡率最高的恶性肿瘤之一，其精准诊疗与药物开发亟需能够高度模拟人体内真实病理生理特征的体外模型。类器官芯片作为一种新兴技术平台，通过微流控工程、生物材料等多种工程技术手段整合类器官培养，能够精确控制细胞微环境，从而在体外高度模拟人体器官的三维结构与生理功能。该类系统在肿瘤研究、发育生物学和疾病建模等领域展现出显著优势，不仅可保留患者样本异质性和病理特征，还支持多种细胞共培养，重构肿瘤微环境（tumor microenvironment, TME）。然而，目前该技术在肺癌研究中的标准化构建方法与整合分析策略尚待完善。本综述系统阐述了类器官芯片的关键技术原理、在肺癌模型构建、药物筛选及免疫治疗研究中的最新进展，旨在为推动其在肺癌精准医疗与转化研究中的深入应用提供理论依据与技术展望。

## Abstract (en)

Lung cancer remains one of the most prevalent and lethal malignancies worldwide. The advancement of its precise diagnosis and therapeutic development urgently requires *in vitro *models that can highly recapitulate the pathophysiological characteristics of human tissues. Organ-on-a-chip has emerged as a novel technological platform that integrates microfluidic engineering, biomaterials, and other engineering strategies with organoid culture. This platform enables precise control over the cellular microenvironment, thereby closely mimicking the three-dimensional structure and physiological functions of human organs *in vitro*. Organ-on-a-chip systems demonstrate significant advantages in cancer research, developmental biology, and disease modeling, as they not only preserve the heterogeneity and pathological features of patient samples but also support co-culture of various cell types to reconstruct the tumor microenvironment (TME). However, standardized construction methods and integrated analytical strategies for this technology in lung cancer research remain to be further refined. This review systematically elaborates on the key technical principles of organ-on-a-chip and its recent advances in lung cancer modeling, drug screening, and immunotherapy research. It aims to provide a theoretical foundation and technical perspective for promoting the deeper application of organ-on-a-chip in precision medicine and translational research for lung cancer.

肺癌为全球发病率与死亡率最高的恶性肿瘤，主要分为小细胞肺癌（small cell lung cancer, SCLC）和非小细胞肺癌（non-small cell lung cancer, NSCLC），后者包括腺癌、鳞状细胞癌、大细胞肺癌及神经内分泌癌等亚型^[1]^。肺癌在基因型和临床表型上均表现出高度异质性。尽管近年来化疗、靶向治疗及免疫治疗等手段不断进步，显著提升了患者疗效与生活质量，但早期诊断困难、侵袭转移机制复杂及化疗耐药等问题仍是临床治疗的主要挑战。与此同时，美国食品药品监督管理局（Food and Drug Administration, FDA）通过“突破性疗法认定”（breakthrough therapy designation, BTD）^[2]^等加速审批通道，鼓励针对难治性肿瘤的新药开发，并强调需同步开发伴随诊断工具。这一导向对临床前模型的预测准确性、标准化及可重复性提出了更明确的要求。中国国家药品监督管理局（National Medical Products Administration, NMPA）也在深化医疗器械审评改革，明确优先为用于恶性肿瘤治疗且具有显著临床优势的高端医疗器械实施创新特别审查。前沿基础研究同样为模型改进指明方向。肺癌谱系可塑性已被识别为驱动肿瘤进展和耐药的关键机制^[3]^，该过程涉及转录因子与表观遗传调控因子相互作用，以及与肿瘤相关巨噬细胞（tumor-associated macrophages, TAMs）、癌症相关成纤维细胞（cancer-associated fibroblasts, CAFs）等微环境组分的复杂动态交互。能够模拟这种动态可塑性的体外模型，已成为深入研究耐药机制和开发对策的迫切需求。因此，构建能够更准确反映患者体内特征的肺癌组织模型，已成为当前肺癌个体化诊疗策略研究的重要方向与技术难点。

目前肺癌研究模型各有优缺点（表1），常用模型中肺癌细胞系模型缺乏细胞间相互作用及个体异质性，早期肺腺癌特有的肺泡塌陷^[4]^、氧气梯度变化等重要特征无法呈现^[5]^；患者来源的动物移植模型受限于建模周期冗长、难以模拟肿瘤细胞与转移前微环境间的动态交互^[6]^如异常血管生成及免疫逃逸等关键环节，且难以模拟早期肿瘤与免疫细胞之间的动态相互作用^[7]^。近年来迅速发展的肿瘤类器官技术通过患者肿瘤细胞构建的三维培养系统，不仅增殖迅速，且能有效维持肿瘤异质性，为个体化治疗提供了可靠模型^[8]^。类器官是一种在体外利用成体干细胞或多能干细胞通过自组织三维培养形成的、具有对应器官关键细胞类型和空间结构的微型组织模型。但其仍存在一定局限性，如免疫细胞与血管细胞存活率低；营养物质输送不均，致使内部细胞缺氧死亡；现有类器官大多采用静态培养，细胞间排列松散，难以观察癌细胞沿支气管扩散的动态过程^[9]^。鉴于二维细胞系、动物移植模型及肿瘤类器官均无法完整再现肺组织的生理特性，为更精准模拟体内微环境，类器官芯片技术应运而生。类器官芯片通过微流控、3D打印等技术构建功能化血管通道，可模拟肺组织呼吸时的周期性形变^[10]^，并广泛应用于基础医学研究。

**表1:** 不同研究模型的优缺点

Characteristics	Cancer cell line	Animal model	Organoid model	Organoid chip model
Tumor heterogeneity	Low: simulates single cell type only; no TME	High: preserves heterogeneity; cross-species divergence	Moderate: partial heterogeneity; incomplete TME	High: faithfully maintains heterogeneity and TME
Microenvironment simulation	None: lacks stroma/immune cells/vasculature	High: physiological milieu; poor control	Moderate: dominantly malignant cells	High: integrated vasculature, immune crosstalk
Monitoring	Low: static culture; no dynamic observation	Moderate: longitudinal imaging; limited resolution	Moderate: fixed-timepoint imaging	High: continuous live-cell tracking
HTS	High: scalable; low clinical translatability	Low: resource-intensive; incompatible with HTS	Moderate: semi-automated; moderate throughput	High: automation-compatible for large-scale HTS
Drug test	Low: no TME; poor clinical relevance	Moderate: affected by species-specific pharmacokinetics	Moderate: improved over cancer cell line	High: high drug response and clinical consistency
Cost and cycle	Low cost; days-weeks	High cost; months-years	Moderate cost; weeks	Moderate cost; days-weeks
Technical complexity	Low: basic cell culture	High: demands specialized facilities and animal handling skills	Moderate: advanced 3D culture	High: microengineering and biosensing
Limitation	Non-physiological TME; low predictivity	Non-human microenvironment; ethical constraints	Absent dynamic flow; simplified TME	Elevated cost; ongoing standardization challenges

TME: tumor microenvironment; HTS: high-throughput screening; 3D: three-dimensional.

类器官芯片是对传统类器官培养的系统升级，在保留类器官原有优势的基础上，融合微流控与组织工程方法，实现对细胞微环境的精确调控，再现器官特异性微观结构与功能，为精准医学提供更加可靠的研究平台。该类芯片可支持多种细胞类型的长期共培养，通过微腔、多孔膜和力学加载装置模拟体内营养/氧气输送和呼吸相关的机械微环境，显著增强模型生理相关性。同时，该技术具备高通量、自动化潜力，能够实时监测肿瘤演进和生物标志物动态变化，如表皮生长因子受体（epidermal growth factor receptor, *EGFR*）^[11]^、间变性淋巴瘤激酶（anaplastic lymphoma kinase, *ALK*）、Kirsten大鼠肉瘤病毒癌基因同源物（Kirsten rat sarcoma viral oncogene homolog, *KRAS*）突变及药物反应，准确评估药效与毒性，在抗肿瘤药物筛选与个体化治疗中展现出广阔的应用前景。相较于传统静态培养难以模拟体内动态压力环境^[9]^，微流控技术通过精确调控微小空间内的温度、压力等参数，可有效模拟肿瘤微环境（tumor mircroenvironment, TME）的复杂特征，并能重现肺泡在病原体攻击下的免疫反应过程，结合高清显微成像实现机械刺激下细胞动态的实时观测^[12]^。

本文系统综述了类器官芯片技术在肺癌早期诊断、疾病进展及治疗中的应用与研究进展（表2）^[13-26]^。

**表2:** 类器官芯片技术在肺癌研究中的核心应用与代表性研究

Sample source	Technical features	Clinical significance
Peripheral blood	In-situ capture and ex vivo expansion of CTCs	Provides a powerful tool for non-invasive early diagnosis of lung cancer via liquid biopsy and monitoring of treatment efficacy^[13]^
Peripheral blood	Co-cultivation of autologous immune cells and CTCs in micro-wells, combined with multi-drug concentration gradient screening	Enables individualized drug sensitivity assessment and provides references for clinical medication regimens^[14]^
Peripheral blood	Using a microfluidic platform and nanopore electrical delivery technology to achieve in-situ quantitative analysis of multiple genes and synchronous phenotyping at the single-cell level, and construct an ICI efficacy prediction index	Constructs an ICI efficacy prediction index to guide clinical precise immunotherapy^[15]^
A549 cell line	Co-cultivation of A549/HUVECs on a microfluidic chip and delivery of miR-497 exosomes	Investigates the mechanism of exosome-mediated tumor-vascular crosstalk and provides a new strategy for anti-angiogenic therapy^[16]^
AT2	Isolate patient-derived AT2 cells and combine them with feeder-free organoid cultures for AT2 expansion while preserving the expression of their markers	It provides an unprecedented research platform for revealing the malignant transformation of AT2 induced by oncogenic mutations, and greatly advances the understanding of the initiation stage of lung adenocarcinoma^[17]^
Patient-derived tumor cells	Development of a Transwell-integrated organoid chip platform simulating tumor metastasis	Offers a powerful tool to address the therapeutic challenges posed by the heterogeneity of metastatic lesions^[18]^
A549 cell line	Development of a 3D-cultured multi-organ microfluidic platform with precisely controllable dissolved oxygen concentration	Simulates the tumor hypoxic microenvironment to study hypoxia-induced metastasis mechanisms and drug responses^[19]^
A549 cell line	Construction of a lung cancer bone metastasis chip to enable visualization of the bone metastasis process	Provides a visual platform for researching bone metastasis mechanisms and developing anti-metastatic drugs^[20]^
Patient-derived tumor cells	Development of an InSMAR-chip that enables drug sensitivity testing of lung cancer organoids within approximately 1 week using an ultralow cell number	Provides a rapid, miniaturized platform for drug response prediction, which maintains high genetic consistency with the original tumor to ensure reliable outcomes^[21]^
Patient-derived tumor cells	Development of a fully humanized MIRO platform to model the spatial organization of the tumor-stroma interface and its interaction with immune cells	Serves as a powerful tool for immunotherapy testing, predicting patient-specific responses to immunomodulatory therapies, and provides insights into overcoming stromal-mediated immunosuppression and treatment resistance^[22]^
Patient-derived tumor cells	Proposal of an innovative vascularized patient-derived tumor organoid chip with a hierarchical tumor-specific microvascular system	Replicates the complex intercellular crosstalk in the tumor microenvironment and provides a new perspective for studying angiogenesis and metastatic heterogeneity^[23]^
Patient-derived tumor cells	Optimization of cell interactions via a superhydrophobic micro-well chip and establishment of a gel-liquid interface co-culture model of lung cancer organoids and matched PBMCs	Dynamically tracks T-cell differentiation and drug responses, reveals immune synergy mechanisms, and provides an efficient predictive platform for individualized immunotherapy^[24]^
Patient-derived lung tumor	Lung tumor-on-chip with live imaging to quantify anti-PD-1 effects	Evaluate personalized anti-PD-1 response and matrix resistance mechanisms^[25]^
Patient-derived tumor cells	Development of a functionally relevant scRNA-seq platform that allows phenotypic assessment and scRNA-seq at the single organoid level	Provides a multifunctional research platform for evaluating novel immunotherapies and analyzing molecular mechanisms^[26]^

CTCs: circulating tumor cells; ICI: immune checkpoint inhibitor; HUVECs: human umbilical vein endothelial cells; AT2: alveolar epithelial type II cells; InSMAR-chip: integrated superhydrophobic microwell array chip; MIRO: micro immune response on-chip; PBMC: peripheral blood mononuclear cells; scRNA-seq: single-cell RNA sequencing; PD-1: programmed cell death protein 1.

## 1 类器官芯片概述

类器官芯片技术通过整合微流控、3D生物打印等工程方法，已成为体外模拟器官生理与病理过程的重要平台。微流控技术指在微米级通道中精确操控微量流体，其在类器官芯片中通过微泵、微阀等流体控制系统，动态调节培养腔内的流体剪切力、营养物质及气体交换。3D生物打印技术的引入极大改善了血管模型的结构完整性。其打印出的三维血管网络具备内皮屏障功能。类器官芯片技术显著提高了类器官培养的稳定性、仿生性及实验可重复性，为高度模拟人体器官复杂结构与功能提供了关键技术支持。

类器官芯片通常由上下两层微管道系统构成。上层用于培养肺泡上皮细胞，形成气-液界面；下层则培养血管内皮细胞，构建微型血管网络。二者通过多孔薄膜分隔，模拟肺泡-毛细血管屏障结构与功能特征（图1）^[27]^。芯片侧缘集成模拟呼吸运动的机械装置，研究者将不同细胞分别植入对应微管，持续灌注专属培养基。经培养后，细胞可分化为具有实际功能的组织单元^[1]^。在材料方面，聚二甲基硅氧烷（polydimethylsiloxane, PDMS）因其良好的气体通透性与光学透明度被广泛采用^[28-30]^。但其疏水性和小分子吸收性限制了其在药物渗透研究中的应用。

**图1:**
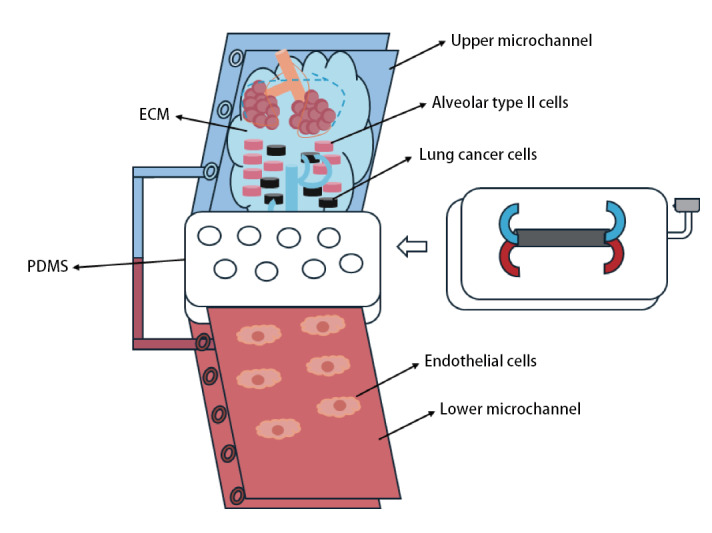
肺癌类器官芯片概述图

类器官芯片的功能实现高度依赖于其核心培养基质，而不同基质在其应用特点上性能表现各异（表3）。基质胶（Matrigel basement membrane matrix, Matrigel）是应用最为广泛的基质材料，其主要成分包括层粘连蛋白、IV型胶原和巢蛋白^[31]^，能较好地支持类器官的出芽、极性和增殖。在基础研究中，其表现出了较高的可靠性，然而，Matrigel存在显著的临床转化障碍。首先它来源于Engelbreth-Holm-Swarm小鼠肉瘤^[31]^，成分复杂（包含超过1800种未明确蛋白质），存在潜在外源病毒污染风险以及引发外来免疫原性的问题。目前Matrigel主要被批准用于科研领域，尚未获得《药品生产质量管理规范》级别认证，难以满足临床治疗产品对成分明确性和批次一致性的严苛要求^[32]^。为克服Matrigel的局限，研究者引入水凝胶^[33]^与光固化树脂^[34]^等新型材料的应用，使体外模型更贴近体内真实反应，可实现精准药效评估，包括多种新型培养体系如纤维蛋白原-凝血酶水凝胶^[35]^，具有良好的生物活性和人源性优势，存在力学可调范围窄、易降解的问题；甲基丙烯酰化明胶和甲基丙烯酰化透明质酸等合成水凝胶，具备力学性能可编程（模量通常在1-50 kPa内可调）^[36]^、批次间一致性高的优点。

**表3:** 类器官培养用不同基质类型的优缺点

Matrix type	Advantages	Limitations
Matrigel	Widely used, well supports organoid budding, polarity and proliferation; demonstrates high reliability in basic research	Programmable mechanical properties, high batch consistency, clear composition, and conducive to standardization and clinical translation
Natural hydrogel	Possesses good biocompatibility and human-derived advantages	Narrow adjustable range of mechanical properties; prone to degradation and poor stability
Synthetic hydrogel	Programmable mechanical properties, high batch consistency, clear composition, and conducive to standardization and clinical translation	Generally lower bioactivity than natural matrices; requires the introduction of active peptide segments or functional modification

## 2 类器官芯片推动肺癌研究发展

### 2.1 面向肺癌早期诊断与临床转化的液体活检新策略

液体活检通过检测血液中的癌细胞或肿瘤DNA来诊断和解析肺癌。虽然微流控技术能高效富集循环肿瘤细胞（circulating tumor cells, CTCs）、外泌体等标志物，但其传统平台仅限于分离和计数，缺乏对标志物生物学功能的验证。类器官芯片技术解决了这一局限，它将微流控的高效捕获与类器官培养相结合，不仅能获取标志物，还能直接进行功能验证，从而显著提高了液体活检的临床转化价值。

#### 2.1.1 CTCs在肺癌早期诊断与个体化治疗中的应用进展

CTCs是指从原发性或转移性病灶脱落，进入外周血的肿瘤细胞。CTCs分析方便、无创、高重复性，有利于肺癌早期诊断标志物的筛查。但外周血中CTCs的浓度低于其他血细胞（比例约为1:109），且形态异质性显著^[37]^。近年来研究者^[38]^通过微流控系统高精度流量控制多样品自动处理实现了对CTCs的高效捕获，但难以维持细胞活性及后续扩增，无法深入探究其转移潜能及药物敏感性。类器官芯片平台通过将CTCs捕获模块与类器官培养微环境相整合，实现了从“捕获”到“培养”再到“分析”的一体化流程。研究团队开发了一种基于微流控技术的CTCs与成纤维细胞原位共培养类器官模型。该模型不仅能够保留肿瘤特异性标志物，其所携带的*TP53*突变也与原发肿瘤高度一致，从而为基因表达、蛋白质功能及表型特征等多个层面的深入分析提供了可靠平台^[13]^。在此基础上，有研究者^[14]^利用源自患者血液样本的CTCs，在类器官芯片平台上构建体外肿瘤模型，进而实现对不同治疗策略反应的精准预测，为推进肺癌个体化治疗提供了强有力的技术支撑。

在临床转化方面，有研究者^[15]^采用独特的纳米孔增强电递送系统，能够高效、快速地将稳定的多通道荧光探针导入CTCs中，实现对靶基因表达的原位定量。同时，通过CTCs与免疫细胞在芯片上的共培养，可实时关联并定量其在免疫检查点抑制剂处理下的表型异质性用于筛选可能从免疫治疗中获益的患者。值得注意的是在NSCLC的精准治疗中，CTCs所表现出的耐药表型与下一代测序（next-generation sequencing, NGS）基因检测显示的敏感突变不一致，是临床决策中的常见难题。这种基因型与表型分离的主要根源在于肿瘤异质性。研究表明，早期NSCLC患者的原发肿瘤组织与CTCs之间的基因突变一致率较低（例如一项研究仅显示17.8%），说明肿瘤内部存在多种遗传背景不同的亚克隆。这些亚克隆广泛分布于原发灶、转移灶和循环系统中，其中某些低频耐药克隆可能因变异频率低于NGS检测限而被遗漏^[39]^。除遗传异质性外，非遗传机制也在CTCs耐药中发挥关键作用。TME可通过细胞因子激活信号转导与转录激活因子3（signal transducer and activator of transcription 3, STAT3）、磷脂酰肌醇3-激酶/蛋白激酶B（phosphatidylinositol 3-kinase/protein kinase B, PI3K/AKT）等信号通路，不依赖基因突变即可诱导耐药表型^[40]^。此外，CTCs与血小板或免疫细胞形成簇^[41]^、低氧微环境诱导缺氧诱导因子-1α（hypoxia-inducible factor-1 alpha, HIF-1α）上调、流体剪切力触发上皮-间质转化（epithelial-mesenchymal transition, EMT）^[42]^及细胞骨架重排等，均可导致多药耐药，而这些变化无法通过DNA测序识别。为解决这一困境，多技术联合策略已成为重要方向。例如，2025年同济大学陈昶团队^[43]^基于人工智能构建了“clinic-RadmC”多组学模型，整合临床特征、影像组学及循环游离DNA（circulating cell-free DNA, cfDNA）片段组学数据，为非侵入性肺癌诊断与治疗决策提供了新工具。

#### 2.1.2 外泌体检测在肺癌早期筛查与分型中的转化价值

外泌体作为肿瘤细胞与微环境通信的关键载体，其功能研究需在复杂的生物学背景下进行。近年来，基于微流控技术的外泌体分离与检测取得了重要突破，为肺癌的早期诊断与精准分型提供了新的途径。例如，微流控技术联合高亲和力适配体实现了对肺癌来源外泌体膜蛋白的超灵敏检测，其能够选择性地从NSCLC患者中分离和释放比健康供体多76%的外泌体。也有研究者^[44,45]^在此基础上实现8 min即可从微量血液样本（<300 μL）中获取外泌体，真正实现一步法外泌体分离纯化。进一步的临床样本（*n*=30）验证^[46]^显示，该系统对肺癌的诊断准确率达到91%（95%CI: 79%-96%），其受试者工作特征曲线下面积（area under the curve, AUC）为0.9378，显著优于传统外泌体酶联免疫吸附法的0.8733，显示出更高的鉴别能力。此外，深度学习驱动的微流控-表面增强拉曼光谱技术能够无标记、高通量地分析外泌体表面蛋白及分子谱，从而准确区分NSCLC的不同亚型（如腺癌与鳞癌），体现出良好的临床应用潜力。

将外泌体分析与类器官芯片技术相结合，为癌症治疗的预测提供了新的研究方向。在一项研究^[16]^中，外泌体被用作微小RNA（microRNA, miRNA）的递送载体，实验证实miRNA-497能够显著抑制在类器官芯片中培养的NSCLC的肿瘤增殖与血管生成。该类整合系统不仅深入揭示了外泌体作为生物标志物的作用机制，也推动了液体活检从传统的“标志物检测”向“功能验证”转变，极大提升了肺癌早期诊断的准确性及临床转化价值。

### 2.2 类器官芯片揭示肺癌早期发生与发展机制

作为肺癌的主要病理类型之一，肺腺癌的早期起源与驱动机制一直以来都是研究的难点。类器官芯片技术在揭示致癌突变引发肺泡2型上皮细胞（alveolar epithelial type II cells, AT2）恶性转化中提供了前所未有的研究平台，极大推动了对肺腺癌启动阶段的理解。相较于传统类器官培养中存在的高失败率与杂质污染问题^[47]^，最近的研究^[17]^表明类器官芯片培养的患者来源AT2细胞可维持更稳定的表型。尤为重要的是，2024年一项研究^[48]^通过成簇的规律间隔的短回文重复序列系统（clustered regularly interspaced short palindromic repeats/CRISPR-associated system 9, CRISPR/Cas9）基因编辑技术在AT2细胞中特异性诱导*KRAS*致癌突变，成功构建出肺腺癌起源模型。该模型表明，在*KRAS*突变驱动的早期肺腺癌发生过程中，AT2细胞并非直接进入自我更新途径，而是首先进入一个短暂、高可塑性的损伤相关过渡状态。研究进一步发现，阻断该状态下肉瘤原癌基因酪氨酸蛋白激酶（sarcoma proto-oncogene tyrosine-protein kinase, SRC）信号通路（例如使用达沙替尼），或联合*KRAS*抑制剂，可在类器官模型中有效抑制肿瘤启动，为肺腺癌的早期干预提供了新策略。

肺癌的发生不仅源于细胞自主性突变，还依赖于与微环境的相互作用。类器官芯片允许共培养AT2细胞、成纤维细胞、免疫细胞等多种细胞类型，从而揭示细胞间“对话”在癌变中的关键作用。研究^[49]^表明，活化的基质细胞通过分泌特定信号蛋白，如转化生长因子-β（transforming growth factor-beta, TGF-β），促使AT2细胞发生形态转变，加速癌细胞向周围组织渗透。这种技术突破为建立可靠的实验模型奠定基础，对制定个性化治疗方案具有重要价值。

### 2.3 类器官芯片模拟肺癌远端转移的关键过程与机制

肺癌远端转移涉及肿瘤细胞与局部及远处微环境的复杂相互作用。类器官芯片技术能够高度模拟体内条件，通过构建可控的多器官微环境，为研究器官特异性转移机制和开发治疗策略提供了重要工具。

在模型构建方面，首先有研究者^[18]^开发了一种模拟肿瘤转移的Transwell集成类器官芯片平台，从而可以评估水平方向的肿瘤转移；其次研究者将类器官芯片通过微流控系统实现多器官的串联或并联耦合，从而精准模拟肺癌细胞的转移与定植过程。有团队^[19]^开发了一种可精确调控溶解氧浓度的三维培养多器官微流控平台，通过建立常氧/缺氧条件下器官水平的肺癌-肝脏连锁模型，实现了对肿瘤转移过程中氧微环境影响的动态模拟与分析。

在机制研究层面，基于微流控与组织工程技术构建的肺癌骨转移仿生芯片，可模拟体内转移过程中的多个关键步骤，尤其适用于肿瘤转移前微环境（pre-metastatic niche, PMN）形成机制的研究。研究者^[20]^通过构建肺癌骨转移仿生芯片，实现骨转移过程的可视化。研究表明肿瘤条件培养基能够促进破骨前体细胞向破骨细胞分化，并招募巨噬细胞极化为M2型，从而营造有利于肿瘤定植与克隆增殖的免疫抑制微环境。此外，肿瘤条件培养基还会引起转移前骨生长微环境的紊乱，表现为破骨细胞活性增强、M2型巨噬细胞增多，以及松质骨分解代谢亢进，这些变化共同促进了肿瘤细胞的初始播种与后续增殖。骨质过度分解还可释放细胞外基质（extracellular matrix, ECM）中贮存的活性因子与趋化因子，进一步助力肿瘤细胞的迁移与骨定植。

在临床转化方面类器官芯片可用于测试针对特定转移途径的靶向药物，如HIF-1α抑制剂等^[19]^。此外，类器官芯片能够根据患者特异性转移模式定制治疗策略，例如选择血脑屏障穿透性药物^[50]^。同时，根据《NCCN非小细胞肺癌指南》^[51]^和《中国肺癌脑转移诊疗指南》^[52]^的建议，对于发生转移的肺癌患者，应采用多学科综合治疗模式。该模型可预测不同器官转移灶对治疗的反应，从而指导临床用药决策。因此类器官芯片技术为实现真正意义上的个体化精准医疗提供了有力工具，特别是在解决转移灶异质性带来的治疗挑战方面具有独特优势。

## 3 类器官芯片在肺癌抗肿瘤药物筛选中的策略与转化进展

类器官芯片技术通过整合微流控等关键技术，在精准控制流体剪切力、ECM刚度及三维培养环境方面展现出独特优势，从而显著提高了患者来源类器官的培养成功率、一致性与稳定性。例如，有研究团队^[21]^开发了集成超疏水微孔阵列芯片（integrated superhydrophobic microwell array chip, InSMAR-chip），可在约1周内完成肺癌类器官的药物敏感性测试，大幅缩短传统培养所需时间（数周至数月），且所需细胞量极低（仅1000-5000个细胞/芯片单元）。该平台培养的肺癌类器官在肺癌亚型、DNA拷贝数变异、基因突变谱及表达谱方面均与原始肿瘤组织高度一致，即便长期传代后仍保持此特性。而基于常规3D培养的大规模真实世界研究（*n*=107）则报告了83.3%的总体准确率、84.0%的灵敏度和82.8%的特异性^[53]^。尽管InSMAR-chip在数值上显示出更高的预测准确性，但由于两项研究在样本规模、入组患者基线特征及实验操作流程等方面存在较大差异，因此对这些数值结果进行直接比较需谨慎对待。

此外类器官芯片技术构建高通量、高仿真的药物筛选平台，在模拟TME以优化免疫治疗策略的研究中，类器官芯片平台展现出巨大潜力。例如，2025年有研究团队^[22]^开发了微免疫反应芯片（micro-immune response on-chip, MIRO），成功模拟了肿瘤-基质界面的三维空间结构。该平台证实，基质屏障会导致免疫细胞在接近肿瘤时速度减慢、运动受阻，形成物理性免疫排斥，并保护癌细胞免受诸如曲妥珠单抗等靶向治疗所引发的抗体依赖性细胞毒性。研究进一步利用MIRO平台发现，白细胞介素-2（interleukine-2, IL-2）免疫调节能有效提升免疫细胞活力，帮助其突破基质屏障。这一平台为在个性化水平上研究TME如何影响免疫景观及治疗响应提供了强大工具，目前已在乳腺癌、肺癌和结肠癌等实体瘤的研究中展开应用。

综上所述，类器官芯片平台的核心价值在于其能够高效利用微量临床样本（如穿刺或CTCs），通过芯片上的大量技术重复，为个体患者提供统计可靠的药效预测。其分析目标并非推断群体异质性，而是最大化稀有样本价值。在高效排除无效治疗方案的同时，精准聚焦于有潜力的候选药物，最终为临床个体化治疗提供强有力的决策支持。

## 4 类器官芯片在肺癌免疫治疗研究中的构建与应用

### 4.1 血管化类器官芯片模型的构建及其在免疫治疗研究中的应用

类器官血管化，其核心在于构建具有仿生血管网络的三维类器官模型。通过引入微流控芯片和3D生物打印技术，可在类器官中形成功能性的微血管结构，在维持组织代谢稳态和微环境稳定性方面发挥关键作用^[54]^。将血管化整合至类器官芯片技术中，为深入研究相关分子机制及药物筛选提供了更可靠的实验平台。2019年Paek团队^[23]^开发的血管化肺癌芯片具有代表性，他们结合微流控技术和血管自然形成规律，成功测试了化疗药物对血管的损伤效应。但这类早期模型存在明显缺陷：血管仅由单层内皮细胞构成，缺乏保护细胞包裹和基底膜结构，无法反映真实血管对药物渗透的影响。3D生物打印技术的引入极大改善了血管模型的结构完整性。共培养打印血管与类器官不仅可用于研究肿瘤血管生成、转移及药物递送，还可重建免疫细胞浸润过程，评估免疫检查点抑制剂在组织中的分布与药效。2021年Gerigk团队^[55]^研究证实，此类模型可用于研究肿瘤血管生成、转移过程及药物递送效率，并重建免疫细胞浸润过程，评估免疫检查点抑制剂在组织中的分布与药效。在临床转化方面，有研究者^[56]^开发了一种具有灌注和分层脉管系统的血管化类器官。该系统包括接近肿瘤类器官的无组织肿瘤诱导的血管生成和超出发芽区域的有组织微血管网络，研究结果强调了Notch信号在促进癌细胞向新形成的血管网络迁移中的作用并准确地模拟了抗血管生成治疗的临床反应。

虽然3D生物打印技术显著提升了血管模型的结构完整性，但在实际应用中仍存在多方面局限。在材料方面，现有生物墨水难以同时满足打印精度、机械强度及生物相容性要求^[57]^；在技术层面，打印分辨率尚无法模拟真正的毛细血管网络，且多细胞协同打印过程中细胞易受损^[58]^；在功能方面，打印出的血管与周围组织整合不足，屏障功能与体内存在差距，且动态培养中难以精确调控流体环境以长期维持血管功能^[59]^。

尽管存在这些挑战，3D生物打印血管模型仍是目前最接近体内微环境的研究平台，随着多材料打印与动态培养技术的进步，其应用潜力有望进一步释放。

### 4.2 基于类器官芯片的肿瘤免疫微环境（tumor immune microenvironment, TIME）重构与免疫应答研究

类器官芯片模拟TIME的关键在于有效整合功能性免疫细胞。有研究^[24]^表明，从患者外周血分离的单个核细胞或肿瘤浸润淋巴细胞可与肺癌类器官在芯片内共培养，并保持其活性和功能特性。研究者通过凝胶-液体界面模型证实，在抗程序性细胞死亡蛋白1（programmed cell death protein 1, PD-1）治疗后，CD8^+ ^T细胞能够有效浸润肿瘤类器官，分化为效应样肿瘤反应性T细胞，并高表达细胞毒性分子如颗粒酶B和穿孔素。此外，该平台还能模拟免疫抑制细胞的功能，如TAMs和髓源性抑制细胞。通过添加特定细胞因子（如IL-6、IL-10、TGF-β等），研究人员可诱导单核细胞分化为M2型TAMs，再现其抑制T细胞活化的功能。研究^[24]^表明，这些免疫抑制细胞通过多种机制促进免疫逃逸，包括表达程序性死亡配体1（programmed cell death ligand 1, PD-L1）、分泌免疫抑制因子及竞争消耗关键营养素。

TIME的物理化学特征显著影响免疫细胞功能和治疗反应。类器官芯片技术已能够复现TIME中多种生物力学特征，包括基质刚度调控与循环机械拉伸刺激，从而更准确地模拟肺部动态物理微环境。通过在共培养系统中整合免疫细胞、内皮细胞及成纤维细胞，这些平台可实现对细胞直接相互作用与细胞因子介导的间接信号传递的综合建模，为研究肿瘤宏环境与微环境间的动态互作提供了系统性的研究手段^[60]^。

### 4.3 基于类器官芯片的PD-L1动态监测与免疫调控

PD-1受体及PD-L1是调节抗肿瘤免疫反应的关键检查点蛋白^[61]^。近年来，其调控机制研究已从转录水平扩展到蛋白质翻译后修饰（如琥珀酰化）及TME中特定免疫细胞群体的深度调控。在此背景下，类器官芯片平台凭借其整合微流控、活细胞成像与多维检测的优势，为在高度仿生的TME中实现PD-L1的连续、原位监测提供了理想工具。

类器官芯片平台的核心能力在于整合多种技术以实现动态监测。其利用活细胞成像和先进的图像分析算法，能够快速、准确地测量免疫检查点抑制剂对T细胞介导的癌细胞死亡的影响^[25]^。此外，也有研究者^[62]^通过类器官芯片富集非转移性NSCLC患者全血中的CTCs，来监测放疗期间PD-L1的动态变化。研究发现，放疗期间PD-L1表达短暂增加并且CTCs中的PD-L1阳性可能作为接受放疗的非转移性NSCLC患者的预后标志物。

最近的研究^[63]^强调肿瘤衍生的外泌体PD-L1在免疫逃避中的关键作用及其作为诊断和预后生物标志物在癌症免疫疗法中的潜力。基于微流控技术的外泌体捕获与检测系统，可实现无创、连续、高灵敏度的PD-L1定量分析。2025年研究者^[64]^开发了一个集成的微流体平台，实现了PD-L1的原位监测和外泌体介导的T细胞免疫反应。通过创建局部的共培养微环境，可无需直接接触促进外泌体介导的细胞相互作用。通过检测外泌体和细胞因子，从而对原位免疫调节进行空间和定量分析。其证明了PD-L1来自癌细胞的外泌体在邻近的T细胞中显著抑制干扰素-γ（interferon-γ, IFN-γ）和IL-2分泌，从而直接介导免疫抑制。

尽管前景广阔，但将外泌体PD-L1检测系统与肺癌类器官模型进行深度整合的研究仍处于早期阶段。当前工作多集中于二维培养条件下的外泌体-细胞互作，而能够模拟体内复杂三维微环境的“类器官-外泌体PD-L1集成分析平台”尚待开发。如何利用类器官芯片技术，特别是其内置的微流控功能，来构建更完整的免疫微环境模型，并实现对PD-L1等关键靶点的系统生物学分析，无疑是该领域未来突破的重要方向。

### 4.4 类器官芯片结合多组学促进精准免疫治疗

类器官芯片与多组学技术（包括基因组学、转录组学、蛋白质组学和代谢组学）的整合，已成为肿瘤研究领域的重要发展趋势。该策略能够系统解析肿瘤细胞及TME中免疫细胞在基因表达、蛋白合成与代谢活动等多维动态，从而深入揭示肺癌发生发展的分子机制，显著推动肺癌治疗从传统经验模式向多组学驱动的精准干预转变。

该类整合平台具有多方面显著优势：其一，类器官芯片提供高度仿生且可控的微环境，保留了患者特异性的肿瘤异质性和细胞间相互作用，为多组学分析提供了接近体内的理想模型^[24]^；其二，多组学技术可实现在同一群体甚至单细胞水平获取多层次分子信息，极大增强了数据的系统性和完整性，有助于发现新的机制联系和候选靶点。例如，研究人员^[26]^通过对171例原发性肺癌类器官的单细胞转录组测序（single-cell RNA sequencing, scRNA-seq）分析，不仅鉴定了与肿瘤反应性T细胞相关的10个特征基因（如*GNLY*、*CD44*、*CD9*），还提示*CD99*可能作为调控T细胞活性的关键分子，为开发免疫治疗增敏靶点提供了新方向。

然而，该融合策略目前仍面临若干重要局限：多组学数据整合分析与解读对计算方法和生物信息学能力提出极高要求；类器官培养与芯片构建的成本较高、周期较长，一定程度上限制了其临床推广的时效性；此外，如何将多维组学数据与临床表型进行有效关联并转化为可操作的诊疗指标，仍是当前研究的难点。尽管存在挑战，类器官芯片与多组学的结合已展现出巨大潜力。它不仅能高效筛选最优免疫治疗组合，还可在单细胞分辨率下揭示耐药机制（如免疫检查点代偿、代谢重编程和免疫抑制细胞募集等），为最终实现肺癌精准免疫治疗提供强大且不可或缺的工具。

## 5 总结与展望

肺癌研究领域正经历着从传统模型向仿生类器官芯片技术的重大范式转变。传统二维细胞培养无法模拟体内空间结构与微环境异质性，动物模型则存在物种差异及伦理限制，难以精准预测人体内肿瘤行为。类器官芯片技术通过整合微流控系统、3D生物打印、患者来源肿瘤细胞及功能化生物材料实现了多项突破：（1）在模型构建方面，该技术能够构建包含血管网络、免疫细胞、成纤维细胞和ECM的复杂TME，高度重现器官特异性结构、转移前生态位及器官间相互作用；（2）在实时监测方面，结合高分辨活细胞成像、微传感器和外泌体PD-L1动态分析等技术，可实现基因突变追踪、信号通路激活可视化、药物分布及肿瘤-免疫互作的过程监测；（3）在临床转化层面，类器官芯片已展现出辅助早期诊断、筛选有效药物组合及预测治疗反应的巨大潜力，尤其通过3D血管打印构建灌注式功能性血管网络，显著提升营养物质与气体交换效率，延长模型存活时间，进一步提高筛选准确性和个性化治疗指导能力。尽管类器官芯片技术取得显著进展，其临床转化仍面临多个关键挑战：（1）不同实验室在芯片结构设计、材料选择（如水凝胶刚度、降解特性）、培养参数（流速、剪切力、细胞密度）、患者选择、样本来源、培养基配方、药物测试方案以及临床终点定义等方面都存在差异，使得直接比较其结果变得困难且不可靠。亟需推动芯片设计与培养条件的标准化与模块化；（2）血管化能力仍显不足，现有模型中的打印血管多数存活时间短于72 h，缺乏成熟血管的药物屏障功能，需借助3D生物打印技术共打印内皮细胞、周细胞及基底膜组分，构建更稳定、具有生理功能的血管网络；（3）当前芯片制造工艺复杂、成本较高，且适于长期培养的生物墨水种类有限，限制了其大规模临床应用；（4）TME的模拟仍偏简化，特别是TIME中T细胞耗竭、巨噬细胞极化等动态过程尚未充分体现；（5）对外泌体PD-L1等生物标志物的检测灵敏度、标准化操作流程仍有待提高；（6）产生的多组学数据庞大且复杂，如何有效整合并从中提取具有临床指导价值的信息，是目前的一大挑战。

未来，类器官芯片技术的持续创新有望提高晚期患者5年生存率，更快制定个体化治疗方案并大幅降低新药研发成本。为实现这一目标，未来应聚焦以下研究方向：开发标准化、模块化芯片平台；优化血管化类器官与TIME构建工艺；整合人工智能与多组学分析以解析耐药机制；推动外泌体PD-L1等液态活检标志物的临床验证与应用。类器官芯片不仅将深刻改变肺癌基础研究的范式，更有望成为精准医学时代的核心工具。
